# Spinal cord injury and aging: an exploration of the interrelatedness between key psychosocial factors contributing to the process of resilience

**DOI:** 10.1080/21642850.2021.1911656

**Published:** 2021-04-12

**Authors:** Hailey-Thomas Jenkins, Theodore D. Cosco

**Affiliations:** a Department of Gerontology, Simon Fraser University, Burnaby, Canada; b Gerontology Research Center, Department of Gerontology, Simon Fraser University, Burnaby, Canada; c Oxford Institute of Population Ageing, University of Oxford, Oxford, UK

**Keywords:** Resilience, spinal cord injury, aging, social supports, self-efficacy, spirituality

## Abstract

**Context:**

Extant literature highlights how many individuals display resilient trajectories following spinal cord injury (SCI), exhibiting positive psychological adjustment. In the absence of a universal definition, it is agreed that resilience is demonstrated when individuals have better-than-projected outcomes when considering the level of adversity experienced. Previous research has focused on traits connected to vulnerability and maladaptive trajectories following SCI rather than the psychosocial factors that contribute to resilience, which can be cultivated over the lifetime. Individuals living with SCI are now aging and have lifespans paralleling that of the broader older adult population. Aging with SCI can result in a sequela of concomitant pathophysiologic conditions and social challenges, which can undermine resiliency.

**Objective:**

The purpose of the current commentary is to explore some of the psychosocial factors contributing to resilience within the context of aging with SCI.

**Methods:**

Commentary

**Findings:**

Psychosocial factors contributing to resilience within the SCI population include self-efficacy, social supports, and spirituality. However, these factors are complex and their interconnectedness is not well-understood at the intersection of SCI and aging.

**Conclusion:**

Understanding the complexities of the contributing psychosocial factors can allow for the development of targeted and innovative multi-pronged rehabilitative strategies that can support resilient trajectories across the lifetime. Future research should move towards the inclusion of additional psychosocial factors, adopting longitudinal research designs, and prudently selecting methods.

Spinal cord injuries (SCI) profoundly alter the lives of those who sustain them, leading to deleterious psychopathological outcomes if not well-adapted to over the lifetime (Bonanno, Kennedy, Galatzer-Levy, Lude, & Elfström, 2012; Craig, Tran, Guest, & Middleton, 2019; de Carvalho, Andrade, Tavares, & de Freitas, 1998). Maladaptive adjustment to SCI is associated with psychological conditions, such as depression, which can decrease resilience, coping, and quality of life (Bhattarai, Maneewat, & Sae-Sia, 2018; Craig et al., 2019; de Carvalho et al., 1998; Mazur, Sojka, Stachyra-Sokulska, & Lukasiewicz, 2019; White, Driver, & Warren, 2010). For individuals with SCI, ongoing positive psychological adjustment is paramount, as psychopathology can cascade into further physical impairment and functional dependence, compounding existing psychopathologic conditions (Craig & Perry, 2014; Hoffman, Bombardier, Graves, Kalpakjian, & Krause, 2011).

Following SCI, resilience is found to facilitate the successful capacity to reframe one's purpose and reorient to society, leading to positive psychological outcomes (Bonanno et al., 2012; Craig & Perry, 2014; Mazur et al., 2019; White et al., 2010). Resilience is a multi-faceted concept that is defined as ‘the dynamic process of progressing through life challenges to reach a positive developmental outcome or “bounce back,” such as a positive adjustment to significant illness or injury’ (Tansey, Bezyak, Kaya, Ditchman, & Catalano, 2017, p. 163). Rooted in positive and developmental psychology, the concept of resilience forsakes the biomedical tradition of cataloguing adverse psychological outcomes in favour of fostering resources that enhance the capacity for individual resilience (Catalano, Chan, Wilson, Chiu, & Muller, 2011; Quale & Schanke, 2010; Tansey et al., 2017; White et al., 2010). Paradigmatically, resilience shifts research towards inclusivity, resulting in interventions designed for a full-spectrum of individuals with variable functional abilities, and psychosocial resources to draw from (Cosco, Howse, & Brayne, 2017; Tansey et al., 2017; White et al., 2010). Conceptually, resilience is essential to SCI research as it provides the foundation to understand how individuals with SCI can have better than anticipated outcomes, given the significant level of adversity experienced.

Earlier disability discourses posited major disabilities were experienced statically, where future implications were dismissed once the individual had adapted to their injury (Jeppsson Grassman, Holme, Taghizadeh Larsson, & Whitaker, 2012). This problematic discourse overlooks the dynamic and continual challenges of aging with this injury, which can have additive and reciprocal effects (Geard, Kirkevold, Løvstad, & Schanke, 2018). Individuals encounter serious multi-system secondary health conditions which complicate the SCI clinical course by further impairing the body systems (Mortenson et al., 2014). Complex outcomes materialize when SCI, secondary health conditions, and aging are intersected, which can undermine resilience leading to worsening psychopathology, such as the co-occurrence of depression and chronic pain (Mortenson et al., 2014). For those with SCI, the process of aging may increase the vulnerability of developing psychopathological conditions and the ability to remain resilient.

Individuals aging with SCI are an emergent older adult population, and poor psychopathological outcomes are found to be both detrimental and remediable in later life (Mortenson et al., 2014; Segal, Quals, & Smyer, 2018). Ongoing adaptation to aging with SCI is challenging, as physical disability and functional dependence can accrue over time (Duggan, Wilson, DiPonio, Trumpower, & Meade, 2016); however, many individuals living with SCI display resilience and positive psychological outcomes despite advancing demands (Bonanno et al., 2012; Geard et al., 2018; Quale & Schanke, 2010). Current literature demonstrates patterns of resilience and positive psychological outcomes within this population, demonstrating psychopathology is not an inevitable consequence of this injury and aging (Bonanno et al., 2012; Craig et al., 2019; Mortenson et al., 2014; Segal et al., 2018). Psychosocial factors contribute to the process of resiliency among individuals with SCI and are found to include self-efficacy, social supports, and spirituality. By comprehensively understanding the psychosocial factors which contribute to the process of resilience and positive psychological outcomes, there are opportunities to circumvent maladaptive trajectories in older adults with SCI and develop appropriate methods to remediate them if they do occur.

Strong self-efficacy and social supports, are distinct key psychosocial contributors amongst resilient SCI trajectories, informed by individual constitutional factors. Although categorically distinct contributors, the functional support required to live with SCI causes an interrelatedness amongst these key psychosocial contributors, in which they operate as cyclical influencers of each other, and resilience (Bhattarai et al., 2018; Catalano et al., 2011; Duggan et al., 2016; Geard et al., 2018; Mazur et al., 2019; Monden et al., 2014). The cyclic effects between social supports and self-efficacy are evidenced in both maladaptive and resilient trajectories (Duggan et al., 2016; Geard et al., 2018; Kornhaber, Mclean, Betihavas, & Cleary, 2018). As an independent key contributor, strong self-efficacy early on is predictive of both early and ongoing resilience in overcoming the challenges of SCI and maintaining positive psychological outcomes (Bhattarai et al., 2018; Mazur et al., 2019; Tansey et al., 2017). The relative importance of these key contributors and their interrelatedness is demonstrated by the findings that a higher level of injury severity is not always predictive of worse psychopathologic outcomes and quality of life (de Carvalho et al., 1998; Tansey et al., 2017). As self-efficacy is defined as our personal beliefs as to our capacity to independently carry-out behaviours in relation to goal attainment (Tansey et al., 2017), aging with SCI appears to present a life circumstance that degrades this capacity and the potential for these beliefs. This highlights that even if severe disability undermines the capacity for functional independence, strong self-efficacy and resilience are possible (Tansey et al., 2017). Additionally, it points to the role of an interconnected psychosocial contributing factor, which is social support.

Social supports play an integral role in the daily coping of life with SCI, and the diverse set of social supports can include: family, friends, healthcare workers, and peers with SCI (Duggan et al., 2016; Geard et al., 2018; Monden et al., 2014). The functional and dispositional characteristics of the support itself plays a role in how self-efficacy is subjectively appraised and redefined in the presence of SCI, as do underlying individual constitutional factors (Bhattarai et al., 2018; Catalano et al., 2011; Kornhaber et al., 2018). Constitutional factors can include: premorbid coping strategies, personality traits, and demographics (Catalano et al., 2011; Mazur et al., 2019; Monden et al., 2014). Evidence exists that social supports are both enhancers and barriers to self-efficacy, linked to resilience and psychological outcomes finding some individuals perceiving their requirement for support as burdensome and others, as external sources of motivation to overcome challenges (Duggan et al., 2016; Geard et al., 2018; Monden et al., 2014). Individual premorbid constitutional factors that underlie the process of subjective appraisal of the social support itself may account for the difference in findings. For example, extroverted and optimistic individuals are found to be resilient, exhibiting positive psychologic adjustment, whereas neurotic and less optimistic individuals are found to exhibit depression and thereby less resilience (Bonanno et al., 2012; Campbell-Sills, Cohan, & Stein, 2006; de Carvalho et al., 1998; Quale & Schanke, 2010). The subjective appraisal of self-efficacy and social supports is an ongoing process, linked to fluctuating individual functional capacity and available resources (Duggan et al., 2016; Geard et al., 2018). The cross-sectional design of most studies does not enable us to understand resilience within the context of aging and over the lifetime, rather inadvertently situates us to fail to understand this phenomenon and foster this capacity amongst those who may benefit most from intervention. Ultimately, resilience cannot exist without the psychosocial contributions of self-efficacy or social support in the presence of SCI. Altogether, in the context of aging, this becomes critically important as aging presents a second time in these individuals lives in which resilience is necessary to assist in achieving positive psychological outcomes.

Furthermore, in the context of SCI, spirituality can uniquely contribute to the process of resilience (Jones, Simpson, Dorsett, & Briggs, 2018; Monden et al., 2014; White et al., 2010). Spirituality is defined as ‘a universal and fundamental human quality involving the search for a sense of meaning, purpose, morality, well-being, and profundity in relationships with ourselves, others, and the ultimate reality’ (Canda & Furman, 2009, p. 59). Classically associated with religious faith, this definition extricates this conceptual boundary, positioning spirituality as a broad construct, inclusive of many meaning-making processes (Jones, Simpson, Briggs, & Dorsett, 2016, p. 922). Individuals utilize spirituality to support themselves in overcoming challenges and establish meaning within their relationships and following SCI, social supports are found to be an identified source of spirituality (Canda & Furman, 2009, p. 1; Jones et al., 2018). As a source of spirituality, social supports are ‘tested’ by the SCI, in which the individual with the injury seeks to determine if their pre-injury relationship/s exist in the manner they perceived them to (Jones et al., 2018). Finding that a perceived pre-injury relationship aligns with reality of the relationship following SCI, re-affirms the strength of the relationship and social supports as a source of spirituality which facilitate resilience (Jones et al., 2018). Re-affirming relationship strength was found to mutually benefit both the individual with SCI, and the social support, founded by the ‘deepening of relationships’ (Jones et al., 2018, p. 527). However, this finding is inconclusive as a later study by Jones, Simpson, Briggs, Dorsett, and Anderson (2019) found that no intercorrelation exists between spirituality and resilience of the individual with SCI, and their social supports. While an explanation for the lack of mutual dyadic benefits is not given, the authors note that this finding is inconsistent with similar studies employing qualitative methods (Jones et al., 2019). Spirituality, as an inconclusive psychosocial contributor of resilience warrants further empirical examination.

Spirituality is similarly found to be a psychosocial contributor to resilience in later life amongst the gerontological literature, reflecting a parallel dynamic between individuals with SCI and older adults, who also draw from spirituality to gain a deeper meaning for their life's purpose (Sytsma et al., 2018). The losses associated with aging may echo the earlier losses associated with sustaining SCI and introduce new ones, such as the loss of social support. As social support is fundamental to aging with SCI and these types of care relationships are demanding overtime, spirituality focused interventions may hypothetically assist in sustaining resilient trajectories.

Current literature may be interpreted as demonstrating that older adults with SCI are resilient; however, in the absence of age-specific cohorts, this finding is unable to be corroborated. The reviewed literature does not explicitly examine aging in the context of SCI, and resilience, rather includes older adults in samples with extreme age ranges (i.e. 18–80). As differences are not currently explored between age groups, it is reasonable to conclude that these mixed-age cohorts may not reflect how the psychosocial factors which contribute to resilience unfold amongst those aging with SCI. Across the lifetime, the manner in which resilience is displayed can evolve and be altered by the specific type of hardship encountered (Cosco, Kok, Wister, & Howse, 2019, p. 91) and therefore, the current approach can limit the strength of the conclusions which can be derived and the ability to support resilient trajectories within this emerging population.

Many older adults demonstrate resiliency and they achieve this resiliency by the psychosocial contributions of self-efficacy and social supports. These two psychosocial factors are intertwined in the presence of SCI and have the potential to be synergistic in their effects upon resilience and positive psychological outcomes. Furthermore, spirituality is an under-examined component of resilience that is both a factor in SCI and the gerontological populations. Spirituality must be examined amongst this population, as it may offer the possibility to help re-establish resilience and reinforce the psychosocial contributions of social supports and self-efficacy. Future research should use a longitudinal trajectory approach, incorporating measures of self-efficacy, social supports, and spirituality as it could assist in establishing both the function and predictability of these psychosocial factors in contributing and producing resilient trajectories when aging with SCI. The addition of interrelated factors and measures including appraisals of secondary health conditions and perceived quality of life, which are also found to be related to resilience, could further enhance such studies and offer a more robust understanding of this topic. For example, it was demonstrated that high positive appraisals, low negative appraisals, and a low severity of secondary health conditions were related to having high self-efficacy (Craig et al., 2019) and poor self-efficacy with considerably lower quality of life (Middleton, Tran, & Craig, 2007). Psychosocial factors contributing to resilience are complex and careful consideration should be given to the selected research methods operationalizing resilience, bearing in mind their merits and limitations to ensure they align with the intended output and adopted definition (Cosco et al., 2019). This emergent older adult population will benefit from rehabilitative strategies that align with the paradigmatic assumption that resilience is an ability that everyone possesses, which can be fostered and strengthened (Quale & Schanke, 2010).

## References

[CIT0001] Bhattarai, M., Maneewat, K., & Sae-Sia, W. (2018). Psychosocial factors affecting resilience in Nepalese individuals with earthquake-related spinal cord injury: A cross-sectional study. *BMC Psychiatry*, *18*(60), 1–8. doi:10.1186/s1288-018-1640-z 29499688 PMC5833058

[CIT0002] Bonanno, G. A., Kennedy, P., Galatzer-Levy, I. R., Lude, P., & Elfström, M. L. (2012). Trajectories of resilience, depression, and anxiety following spinal cord injury. *Rehabilitation Psychology*, *57*(3), 236–247. doi:10.1037/a0029256 22946611

[CIT0003] Campbell-Sills, L., Cohan, S. L., & Stein, M. B. (2006). Relationship of resilience to personality, coping, and psychiatric symptoms in young adults. *Behaviour Research and Therapy*, *44*, 589–599. doi:10.1016/j.brat.2005.05.001 15998508

[CIT0004] Canda, E. R., & Furman, L. D. (2009). *Spiritual diversity in social work practice: The heart of helping*. Cary, NC: Oxford University Press.

[CIT0005] Catalano, D., Chan, F., Wilson, L., Chiu, C.-Y., & Muller, V. R. (2011). The buffering effect of resilience on depression among individuals with spinal cord injury: A structural equation model. *Rehabilitation Psychology*, *56*(3), 200–211. doi:10.1037/a0024571 21843016

[CIT0006] Cosco, T. D., Howse, K., & Brayne, C. (2017). Healthy ageing, resilience and wellbeing. *Epidemiology and Psychiatric Sciences*, *26*(6), 579–583. doi:10.1017/S2045796017000324 28679453 PMC6998987

[CIT0007] Cosco, T. D., Kok, A., Wister, A., & Howse, K. (2019). Conceptualising and operationalising resilience in older adults. *Health Psychology and Behavioral Medicine*, *7*(1), 90–104. doi:10.1080/21642850.2019.1593845 PMC8114384 34040841

[CIT0008] Craig, A., & Perry, K. N. (2014). Guide for health professionals on the psychosocial care of adults with spinal cord injury. Retrieved from https://www.aci.health.nsw.gov.au/__data/assets/pdf_file/0019/155233/Guide-Psychosocial-Care.pdf

[CIT0009] Craig, A., Tran, Y., Guest, R., & Middleton, J. (2019). Trajectories of self-efficacy and depressed mood and their relationship in the first 12 months following spinal cord injury. *Archives of Physical Medicine and Rehabilitation*, *100*(3), 441–447. doi:10.1016/j.apmr.2018.07.442 30218640

[CIT0010] de Carvalho, S. A. D., Andrade, M. J., Tavares, M. A., & de Freitas, J. L. S. (1998). Spinal cord injury and psychological response. *General Hospital Psychiatry*, *20*(6), 353–359. doi:10.1016/S0163-8343(98)00047-4 9854647

[CIT0011] Duggan, C., Wilson, C., DiPonio, L., Trumpower, B., & Meade, M. A. (2016). Resilience and happiness after spinal cord injury: A qualitative study. *Topics in Spinal Cord Injury Rehabilitation*, *22*(2), 99–110. doi:10.1310/sci2202-99 29339852 PMC4896325

[CIT0012] Geard, A., Kirkevold, M., Løvstad, M., & Schanke, A.-K. (2018). Exploring narratives of resilience among seven males living with spinal cord injury: A qualitative study. *BMC Psychology*, *6*(1), 1–10. doi:10.1186/s40359-017-0211-2 29301561 PMC5755441

[CIT0013] Hoffman, J. M., Bombardier, C. H., Graves, D. E., Kalpakjian, C. Z., & Krause, J. S. (2011). A longitudinal study of depression from 1 to 5 years after spinal cord injury. *Archives of Physical Medicine and Rehabilitation*, *92*(3), 411–418. doi:10.1016/j.apmr.2010.10.036 21353823

[CIT0014] Jeppsson Grassman, E., Holme, L., Taghizadeh Larsson, A., & Whitaker, A. (2012). A long life with a particular signature: Life course and aging for people with disabilities. *Journal of Gerontological Social Work*, *55*(2), 95–11. doi:10.1080/01634372.2011.633975 22324328

[CIT0015] Jones, K. F., Simpson, G., Briggs, L., Dorsett, P., & Anderson, M. (2019). A study of whether individual and dyadic relations between spirituality and resilience contribute to psychological adjustment among individuals with spinal cord injuries and their family members. *Clinical Rehabilitation*, *33*(9), 1503–1514. doi:10.1177/0269215519845034 31056938

[CIT0016] Jones, K., Simpson, G. K., Briggs, L., & Dorsett, P. (2016). Does spirituality facilitate adjustment and resilience among individuals and families after SCI? *Disability and Rehabilitation*, *38*(10), 921–935. doi:10.3109/09638288.2015.1066884 26190175

[CIT0017] Jones, K. F., Simpson, G., Dorsett, P., & Briggs, L. (2018). Moving forward on the journey: Spirituality and family resilience after spinal cord injury. *Rehabilitation Psychology*, *63*(4), 521–531. doi:10.1037/rep0000229 30024204

[CIT0018] Kornhaber, R., Mclean, L., Betihavas, V., & Cleary, M. (2018). Resilience and the rehabilitation of adult spinal cord injury survivors: A qualitative systematic review. *Journal of Advanced Nursing*, *74*(1), 23–33. doi:10.1111/jan.13396 28726274

[CIT0019] Mazur, A., Sojka, A., Stachyra-Sokulska, A., & Lukasiewicz, J. (2019). The role of individual predispositions in coping with sudden loss of mobility caused by a traffic accident. *ACTA Neuropsychologica*, *17*(2), 151–165.

[CIT0020] Middleton, J., Tran, Y., & Craig, A. (2007). Relationship between quality of life and self-efficacy in persons with spinal cord injuries. *Archives of Physical Medicine and Rehabilitation*, *88*(12), 1643–1648. doi:10.1016/j.apmr.2007.09.001 18047880

[CIT0021] Monden, K. R., Trost, Z., Catalano, D., Garner, A. N., Symcox, J., Driver, S., … Warren, A. M. (2014). Resilience following spinal cord injury: A phenomenological view. *Spinal Cord*, *52*(3), 197–201. doi:10.1038/sc.2013.159 24418959

[CIT0022] Mortenson, W. B., Sakakibara, B. M., Miller, W. C., Wilms, R., Hitzig, S., & Eng, J. J. (2014). Aging following spinal cord injury. In J. J. Eng, R. W. Teasell, W. C. Miller, D. L. Wolfe, A. F. Townson, J. T. C. Hsieh, S. J. Connolly, V. K. Noonan, E. Loh, & A. McIntyre (Eds.), *Spinal cord injury rehabilitation evidence. Version 5.0* (pp. 1–98). Vancouver.

[CIT0023] Quale, A. J., & Schanke, A. K. (2010). Resilience in the face of coping with a severe physical injury: A study of trajectories of adjustment in rehabilitation setting. *Rehabilitation Psychology*, *55*(1), 12–22. doi:10.1037/a0018415 20175630

[CIT0024] Segal, D., Quals, S., & Smyer, M. (2018). *Aging and mental health* (3rd ed.). Hoboken, NJ: Wiley Black.

[CIT0025] Sytsma, T. T., Schmelkin, L. A., Jenkins, S. M., Lovejoy, L. A., Lapid, M. I., & Piderman, K. M. (2018). “Keep the faith”: Spirituality as a contributor to resiliency in five elderly people. *Journal of Religion, Spirituality & Aging*, *30*(4), 314–324. doi:10.1080/15528030.2018.1441095

[CIT0026] Tansey, T. N., Bezyak, J., Kaya, C., Ditchman, N., & Catalano, D. (2017). Resilience and quality of life: An investigation of Kumpfer’s resilience model with persons with spinal cord injuries. *Rehabilitation Counseling Bulletin*, *60*(3), 163–174. doi:10.1177/0034355216655146

[CIT0027] White, B., Driver, S., & Warren, A. M. (2010). Resilience and indicators of adjustment during rehabilitation from a spinal cord injury. *Rehabilitation Psychology*, *55*(1), 23–32. doi:10.1037/a0018451 20175631

